# Transcriptomic analyses of *Pinus koraiensis* under different cold stresses

**DOI:** 10.1186/s12864-019-6401-y

**Published:** 2020-01-03

**Authors:** Fang Wang, Song Chen, Deyang Liang, Guan-Zheng Qu, Su Chen, Xiyang Zhao

**Affiliations:** State Key Laboratory of Tree Genetics and Breeding, Northeast Forestry University, Harbin, People’s Republic of China

**Keywords:** *Pinus koraiensis*, Cold stress, RNA-seq, Differential expressed genes

## Abstract

**Background:**

*Pinus koraiensis* is an evergreen tree species with strong cold resistance. However, the transcriptomic patterns in response to cold stress are poorly understood for *P. koraiensis*. In this study, global transcriptome profiles were generated for *P. koraiensis* under cold stress (− 20 °C) over time by high-throughput sequencing.

**Results:**

More than 763 million clean reads were produced, which assembled into a nonredundant data set of 123,445 unigenes. Among them, 38,905 unigenes had homology with known genes, 18,239 were assigned to 54 gene ontology (GO) categories and 18,909 were assigned to 25 clusters of orthologous groups (COG) categories. Comparison of transcriptomes of *P. koraiensis* seedlings grown at room temperature (20 °C) and low temperature (− 20 °C) revealed 9842 differential expressed genes (DEGs) in the 6 h sample, 9250 in the 24 h sample, and 9697 in the 48 h sample. The number of DEGs in the pairwise comparisons of 6 h, 24 h and 48 h was relatively small. The accuracy of the RNA-seq was validated by analyzing the expression patterns of 12 DEGs by quantitative real-time PCR (qRT-PCR). In this study, 34 DEGs (22 upregulated and 12 downregulated) were involved in the perception and transmission of cold signals, 96 DEGs (41 upregulated and 55 downregulated) encoding 8 transcription factors that regulated cold-related genes expression, and 27 DEGs (17 upregulated and 10 downregulated) were involved in antioxidant mechanisms in response to cold stress. Among them, the expression levels of c63631_g1 (annexin D1), c65620_g1 (alpha-amylase isozyme 3C), c61970_g1 (calcium-binding protein KIC), c51736_g1 (ABA), c58408_g1 (DREB3), c66599_g1 (DREB3), c67548_g2 (SOD), c55044_g1 (CAT), c71938_g2 (CAT) and c11358_g1 (GPX) first increased significantly and then decreased significantly with the extension of stress time.

**Conclusions:**

A large number of DEGs were identified in *P. koraiensis* under cold stress, especially the DEGs involved in the perception and transmission of cold signals, the DEGs encoding TFs related to cold regulation and the DEGs removing ROS in antioxidation mechanisms. The transcriptome and digital expression profiling of *P. koraiensis* could facilitate the understanding of the molecular control mechanism related to cold responses and provide the basis for the molecular breeding of conifers.

## Background

Cold stress is one of the most important abiotic stresses that adversely affects plant growth and development, crop yield and quality, and geographic distribution [1]. Plants have frequently suffered sudden cold stress, such as early or late frost in nature; subsequently, they would enable a diverse set of response mechanisms to protect against damage [2]. The duration of stress is also a test of plant cold tolerance, which involves various cellular response mechanisms [3]. Studies of the mechanisms that improve cold resistance have suggested the importance of a wide range of physiological, biochemical, cellular and molecular processes, and these processes have been associated with the regulation of gene transcription [4].

At present, low-temperature signal transduction has been widely studied, and the clearest pathway was the C-repeat (CRT)-binding factors (CBF) signal pathway, which is also known as the dehydration-responsive element-binding factors 1 (DREB1) signal pathway and is ABA (abscisic acid)-independent [5]. Many transcription factors are involved in this signal pathway, including CBF1 / DREB1B, CBF2 / DREBlC, CBF3 / DREB1A, CBF4 / DREBlD, DREB1E, DREB1F. *ICE1* (Inducer of CBF Expression 1), is located upstream of *CBF*, and together, they jointly regulate the expression of a spectrum of cold-regulated (COR) genes, through CBF binding to the *cis*-acting element (CRT/DRE) that contains a core conserved sequence of CCGAC [6, 7]. The CBF-COR pathway constitutes the predominant cold signaling pathway in plants, and the *CBF* gene is regulated positively by ICE1 (Inducer of CBF Expression 1). However, HOS1 (High Expression of Osmotically Responsive Gene 1) and MYB15 (myeloblastosis 15) negatively regulate the *CBF* genes, which provides a more complete understanding of the complexity of CBF-mediated cold signaling [8–10].

The expression patterns of cold-responsive genes were different for different plant species during exposure to cold [11–13]. Through the research on *Arabidopsis* transcriptome profiling, a total of 306 genes were identified as cold-responsive genes, with 218 genes increasing and 88 genes decreasing, while the studies on *Cassava* reported that 508 transcripts were identified as early cold-responsive genes, in which 319 sequences had functional descriptions [14, 15]. There have been many similar reports [16, 17]. The number of identified genes involved in cold stress response has been increasing, but the function of most genes have not been revealed. Only 12% of the cold-responsive genes were likely regulated by the CBF transcription factor; therefore, it was predicted that there was a CBF-independent pathway to respond to cold stress in plants [14]. However, to date, there have been few studies on the CBF-independent pathway.

Genome-wide transcriptome analysis is a useful strategy for revealing the molecular mechanism of gene expression, and it can improve the efficiency of identifying the genes of interest. RNA-Seq is a high-throughput DNA sequencing approach, which generates a large amount of transcriptome data for both model and non-model species [18]. This approach has been widely used to analyze the cold stress response of many plants. For example, the gene expression patterns were identified in *Arabidopsis* under drought, cold, high-salinity and ABA-treatment conditions [16]. Comparative transcriptome analysis on two tobacco cultivars (cold-tolerant NC567 and cold-sensitive Taiyuan8) showed that the important COR genes were specifically induced during cold stress in NC567 [19]. The transcriptome analysis of sunflower identified the candidate genes involved in response to chilling and salt stresses [20]. *P. koraiensis* is a famous mixed fruit and wood forest with strong cold tolerance [21, 22]. It is the main tree species in the cold temperate zone and contains abundant cold resistance genes. It is an important material for the study of cold hardiness and acquiring cold resistance genes of coniferous tree species. Thus, it is appropriate and valuable to explore the responsive genes under sudden cold stress in *Pinus koraiensis* through transcriptome sequencing.

*P. koraiensis* is an evergreen tree belonging to Pinaceae*, Pinus*, which is mainly distributed in the northeastern part of China, the Korean peninsula, south of the Russian Far East and Honshu, Japan (124°38′ ~ 140°20′ E, 33°50′ ~ 52°40′ N) [23]. *P. koraiensis* has a strong cold resistance, and it can surmount the extreme low temperature of − 40 °C in its natural growth state. Studies have shown that cold stress resulted in an increase or decrease in the abundance of transcripts associated with several metabolic pathways, and the expression data further suggested the involvement of both the CBF-dependent and independent pathways in the cold responses [5, 14]. In this study, seedlings of *P. koraiensis* that show healthy growth at room temperature suddenly underwent low temperature stress at − 20 °C with stress times of 6, 24 and 48 h, which did not experience cold acclimation. Exploring the expression pattern of genes under sudden cold stress and obtaining the differential expressed genes using RNA-seq and digital expression profiling would provide a valuable genetic resource for cold resistance genes of interest in future conifer breeding process.

## Results

### RNA sequencing and de novo assembly

RNA sequencing was used to investigate the transcriptional changes of *P. koraiensis* under cold stress. In total, twelve cDNA libraries were constructed using RNA extracted from *P. koraiensis* needles, which were exposed to low temperature (− 20 °C) for 0 h, 6 h, 24 h and 48 h, respectively. The cDNA libraries were subjected to paired-end (PE) sequencing by the Illumina HiSeq2000 platform. After filtering out low-quality reads, a total of 763,995,954 clean reads were obtained. The clean reads were de novo assembled into contigs using the Trinity program [24]. A total of 150,528 contigs consisting of 182,705,782 bp, with N50 length of 1951 bp, were obtained. Based on the paired-end sequence information, 123,445 unigenes consisting of 137,320,368 bp with N50 length of 1778 bp were obtained. The expressional levels of the unigenes were obtained by mapping the sequencing reads to the unigenes and normalized using RPKM (reads per kilobase per million mapped reads) method [25].

### Functional annotation of unigenes

**Fig. 1:**
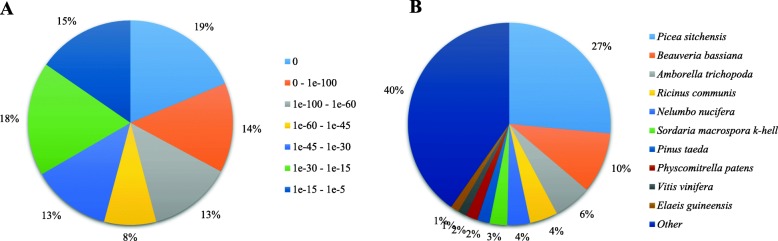
Characteristics of homology search of unigenes against NCBI nonredundant (Nr) database. **a** *E*-value distribution of BLAST hits for each unique sequence with a cut-off *E*-value of 1e^− 5^. **b** Species distribution of the top BLAST hits for each unigene with a cut-off *E*-value of 1e^− 5^

The unigene sequences were mapped to public databases using BLASTn or BLASTx with a cut-off of *E*-value of 10^− 5^. The databases used included NCBI Nucleotide sequence database (Nt), NCBI nonredundant database (Nr), the Universal protein (UniProt) database, the Clusters of Orthologous Groups of proteins (COG) database, the Protein families (Pfam) database, the evolutionary genealogy of genes: Nonsupervised Orthologous Groups (eggNOG) database, the Gene Ontology (GO) database, and Kyoto Encyclopedia of Genes and Genomes (KEGG) database. In total, 38,905 unigenes (31.52%) could be matched to a sequence in at least one of the databases mentioned above. The number of unigenes hits (*E*-value <1e^− 5^) was 31,997 (25.92%) and 28,163 (22.81%) in the Nr and UniProt database, respectively, followed by 22,644 (18.34%) in the Pfam database, 18,909 (15.32%) in the COG database, 18,239 (14.78%) in the GO database, 17,684 (14.33%) in the Nt database, 16,963 (13.74%) in the eggNOG database and 14,201 (11.50%) in the KEGG database (Table 1). The *E*-value distribution of annotation based on the Nr database is shown in Fig. 1a, which indicated that the *E*-values of unigenes (46%) ranged from 1e^− 45^ to 1e^− 5^, and a greater number of unigenes (54%) showed an *E*-value <1e^− 45^, which revealed strong homology. According to a search on the Nr database, the unigene sequences had the strongest BLASTx matches with the gene sequences from *Picea sitchensis* (27%), *Beauveria bassiana* (10%), *Amborella trichopoda* (6%), *Ricinus communis* (4%), *Nelumbo nucifera* (4%), *Sordaria macrospora* (3%), *Pinus taeda* (2%), *Physcomitrella patens* (2%), *Vitis vinifera* (1%) and *Elaeis guineensis* (1%) (Fig. 1b).

**Table 1:** List of *P. koraiensis* transcriptome annotations

Public database	No. of unigene hits	Percentage (%)
Nt	17,684	14.33%
Nr	31,997	25.92%
UniProt	28,163	22.81%
COG	18,909	15.32%
Pfam	22,644	18.34%
eggNOG	16,963	13.74%
GO	18,239	14.78%
KEGG	14,201	11.50%
ALL	38,905	31.52%

*Nt* Nucleotide database, *Nr* Nonredundant protein sequence database, *UniProt* Universal protein database, *COG* Cluster of Orthologous Groups of proteins, *Pfam* Protein families database, *eggNOG* evolutionary genealogy of genes: Nonsupervised Orthologous Groups database, *GO* Gene Ontology database, *KEGG* Kyoto Encyclopedia of Genes and Genomes

**Fig. 2:**
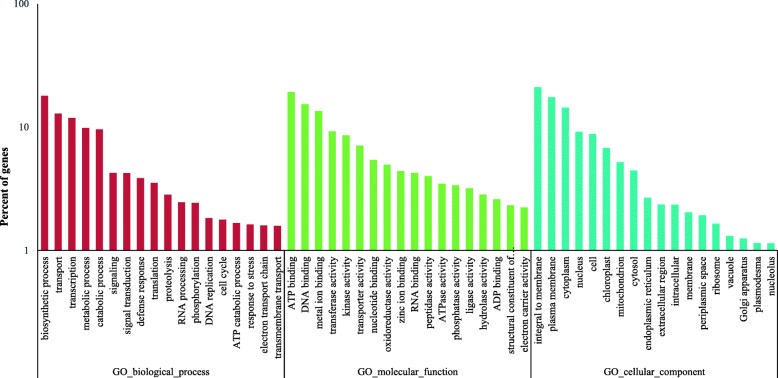
Histogram of Gene Ontology (GO) classifications *in P. koraiensis*

Go analysis was widely used to predict the uncharacterized unigene sequences, and 18,239 unigenes were classified as 54 functional groups. The functional groups were further divided into three categories (‘biological process’, ‘molecular function’, and ‘cellular component’) (Fig. 2) using BLAST2GO [26]. For biological process, most of the unigenes were again classified as ‘biosynthetic process’ (6769), ‘transport’ (4853) and ‘transcription’ (4468); the percentages of total unigenes were 37.11, 26.61 and 24.50%, respectively, followed by ‘metabolic process’ (3705; 20.31%), ‘catabolic process’ (3610; 19.79%). The percentage of others were less than 10%. In the ‘molecular function’ category, the major subcategories were ATP binding (7260; 39.80%), DNA binding (5779; 31.68%) and metal-ion binding (5081; 27.86%), while ‘integral to membrane’ (7942; 43.54%), ‘plasma membrane’ (6593; 36.15%) and ‘cytoplasm’ (5427; 29.75%) were the most representative subcategories in the ‘cellular component’ category (Additional file 1).

**Fig. 3:**
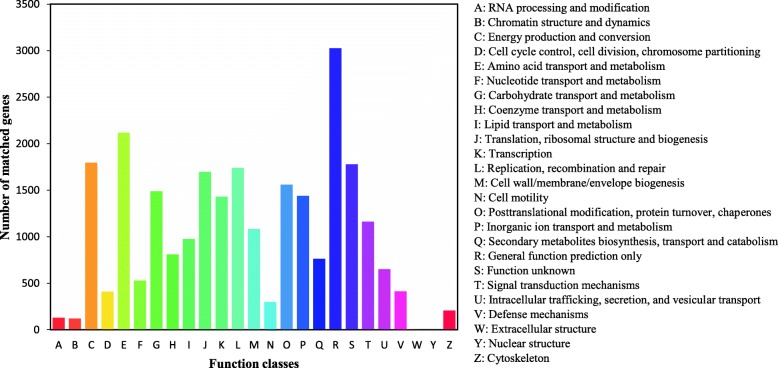
Distribution of unigenes with COG functional classification in the *P. koraiensis* transcriptome

COG database is a protein database. The protein sequences came from the genomes of bacteria, plants and animals. According to the sequence similarity, the 18,909 unigene sequences in *P. koraiensis* were matched to the COG database, which could be grouped into 25 categories (Additional file 2). The top 10 classes were: (R) ‘General function prediction only’ (3025; 16.00%), (E) ‘Amino acid transport and metabolism’ (2117; 11.20%), (C) ‘Energy production and conversion’ (1795; 9.49%), (S) ‘Function unknown’ (1778; 9.40%), (L) ‘Replication, recombination and repair’ (1739; 9.20%), (J) ‘Translation, ribosomal structure and biogenesis’ (1698; 8.98%), (O) ‘Posttranslational modification, protein turnover, chaperones’ (1558; 8.24%), (G) ‘Carbohydrate transport and metabolism’ (1488; 7.66%), (P) ‘Inorganic ion transport and metabolism’ (1439; 7.61%) and (K) ‘Transcription’ (1429; 7.56%). However, the smallest classes were (W) ‘Extracellular structures’ (4; 0.02%) and (Y) ‘Nuclear structure’ (1; 0.01%) (Fig. 3; Additional file 2).

### Differential expressed genes (DEGs)

DEGseq [27] was used to identify differential expressed genes (DEGs) in cold treatment groups in comparison to control group with specified thresholds. In total, 9842, 9250 and 9697 genes were differentially expressed when exposed to cold for 6 h, 24 h and 48 h, respectively. Of the DEGs, 5444, 5241 and 5286 genes were upregulated, while 4398, 4009 and 4411 genes were downregulated at each time point. In addition to identification of DEGs under cold stress, we also identified genes differentially expressed between different time points of cold stress. The numbers of DEGs in the pairwise comparisons of the 6 h, 24 h and 48 h samples was relatively small. A total of 1516 genes were upregulated and 1286 genes were downregulated in the comparison between the 24 h and 6 h samples, 1784 genes were upregulated and 1589 genes were downregulated in the comparison between the 48 h and 6 h samples, and 1364 genes were upregulated and 1226 genes were downregulated in the comparison between the 48 h and 24 h samples, respectively (Table 2).

**Table 2:** The number of DEGs in different comparison groups for *P. koraiensis*

Name	−20 °C 6 h vs CK	−20 °C 24 h vs CK	−20 °C 48 h vs CK	−20 °C 24 h vs − 20 °C 6 h	− 20 °C 48 h vs − 20 °C 6 h	−20 °C 48 h vs − 20 °C 24 h
Up	5444	5241	5286	1516	1784	1364
Down	4398	4009	4411	1286	1589	1226
Total	9842	9250	9697	2802	3373	2590

### Gene ontology analysis of DEGs

GO enrichment was performed to investigate the function of DEGs. GO terms with corrected *P-*values < 0.05 were identified as significantly enriched. In total, 484, 371 and 543 GO terms were significantly enriched after cold treatment of 6 h, 24 h and 48 h, respectively. The top 30 enriched GO terms were shown in Additional file 3. We investigated the enriched GO terms at each time point and found that the enriched biological events of 6 h were similar to 24 h. However, the enriched biological events of 48 h were different from 6 h and 24 h. At time points 6 h and 24 h, the enriched biological processes include ‘response to acid chemical’, ‘response to biotic stimulus’, ‘response to stress’, ‘defense response’, and ‘single-organism developmental process’. The result indicated that plenty of DEGs were involved in the perception and transmission of cold signals. Meanwhile, the enriched molecular functions include ‘kinase activity’, ‘protein kinase activity’, ‘ADP binding’, ‘phosphotransferase activity, alcohol group as acceptor’ and ‘protein serine/threonine kinase activity’, which suggested that the relevant enzyme activities and products might change under cold stress. These results showed a complex regulatory cold stress response and indicated that the changes in the biological process might be very important in response to cold stress in *P. koraiensis*.

### Pathway enrichment analysis of DEGs

KEGG analysis of the DEGs was showed in Additional file 4. The result showed that a total of 46 pathways were significantly affected under cold stress (Q-value < 0.05) in the 6 h sample, among which the top four abundant pathways were ‘plant-pathogen interaction’, ‘plant hormone signal transduction’, ‘starch and sucrose metabolism’ and ‘phenylpropanoid biosynthesis’, followed by ‘protein processing in endoplasmic reticulum’, ‘hippo signaling pathway’ and ‘spliceosome’. For either the 24 h sample or 48 h sample, the top four pathways were the same as those described above. The DEGs in the same pathways in all three stress periods should be of great concern. However, in the pairwise comparison among the 24 h, 6 h and 48 h samples, ‘plant-pathogen interaction’ and ‘phenylpropanoid biosynthesis’ were both included in the top four abundant pathways. Interestingly, ‘metabolism of xenobiotics by cytochrome P450’ could also be noted, because it was enriched in each comparison group.

### Quantitative real-time PCR (qRT-PCR) analysis

**Fig. 4:**
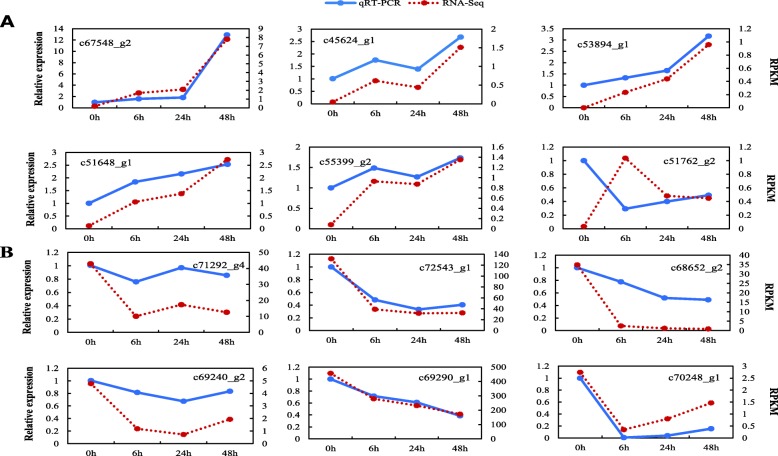
qRT-PCR analysis of DEGs in *P. koraiensis* under cold stress. Transcript levels of 12 randomly selected DEGs, including 6 upregulated (**a**) and 6 downregulated (**b**). The Y-axis on the left shows the relative gene expression levels (2^-ΔΔCt^) analyzed by qRT-PCR (blue lines), while Y-axis on the right shows corresponding expression data of RNA-seq (red dotted lines). The X-axis represents the time (hours) of − 20 °C treatment

To verify the accuracy of the RNA-Seq data under cold stress in *P. koraiensis*, 12 randomly selected DEGs (6 upregulated and 6 downregulated) were used for qRT-PCR analysis of transcript abundance with specific primers (Table 3). The result showed that the expression patterns of 11 of 12 unigenes detected via qRT-PCR were highly consistent with the RNA-Seq result; only c51762_g2 was specific (Fig. 4), which suggested that the high-throughput RNA-Seq data was reliable and demonstrated that the DEGs identified based on transcriptome sequencing were available.

**Table 3:** Primer sequences of *P. koraiensis*

Unigenes	Forward primer	Reverse primer
TUBA	CCAGTTTGTTGATTGGTGTCC	ACGGCTCTCTGAACCTTGG
c67548_g2	GCCTTCGTTCTGCAAGATTTGTCG	CTCACAGCCTTCACAGTCCATT
c45624_g1	TTACAGCACCACCGATTGGAAAGC	GCTGCGATAATCCGCACACTCTT
c53894_g1	AAACTCTGTGTGAGAAGCCGTG	GCATCCCATTCTGGCGACAAA
c51648_g1	TGTGATATACAGTCAGCGGCTGC	CACAGATCCAATCGCAGTTCCA
c55399_g2	AATTTCAAGTTCACTCCGCGCCTC	GTCTGAGCAATATCCAACGGCT
c51762_g2	CTGTATTTGATGCACTTGCCCTGTC	CAATGTGACCAAGAGCCAAGGCAA
c71292_g4	ACCAATCCATCGCCAACAGCAAAG	CACAACCGAAGGATACAACACCCA
c72543_g1	AACACCTGTCACTCCAGAATGCTC	CTATCGACCATGCTGATTTCACCCG
c68652_g2	TAATTTGGTTGCCGAAGCCTGG	CAAAGCTCTGCCCTGTTTCCACAT
c69240_g2	AAGATGTAGTGGTCAGCGAGTGC	GCAAGAGATCGAAACGCTCAAGACA
c69290_g1	ACCTCCGTCTCCGATAATTGAACC	GGAGGCTTAAAGACCAGGAGAGGA
c70248_g1	GATCGAGTTGTGTGTCTGCTTGTG	CCTCTCATGGCTATCTGTTCTCCG

### Gene expression associated with perception and transmission of cold signals in *P. koraiensis*

**Fig. 5:**
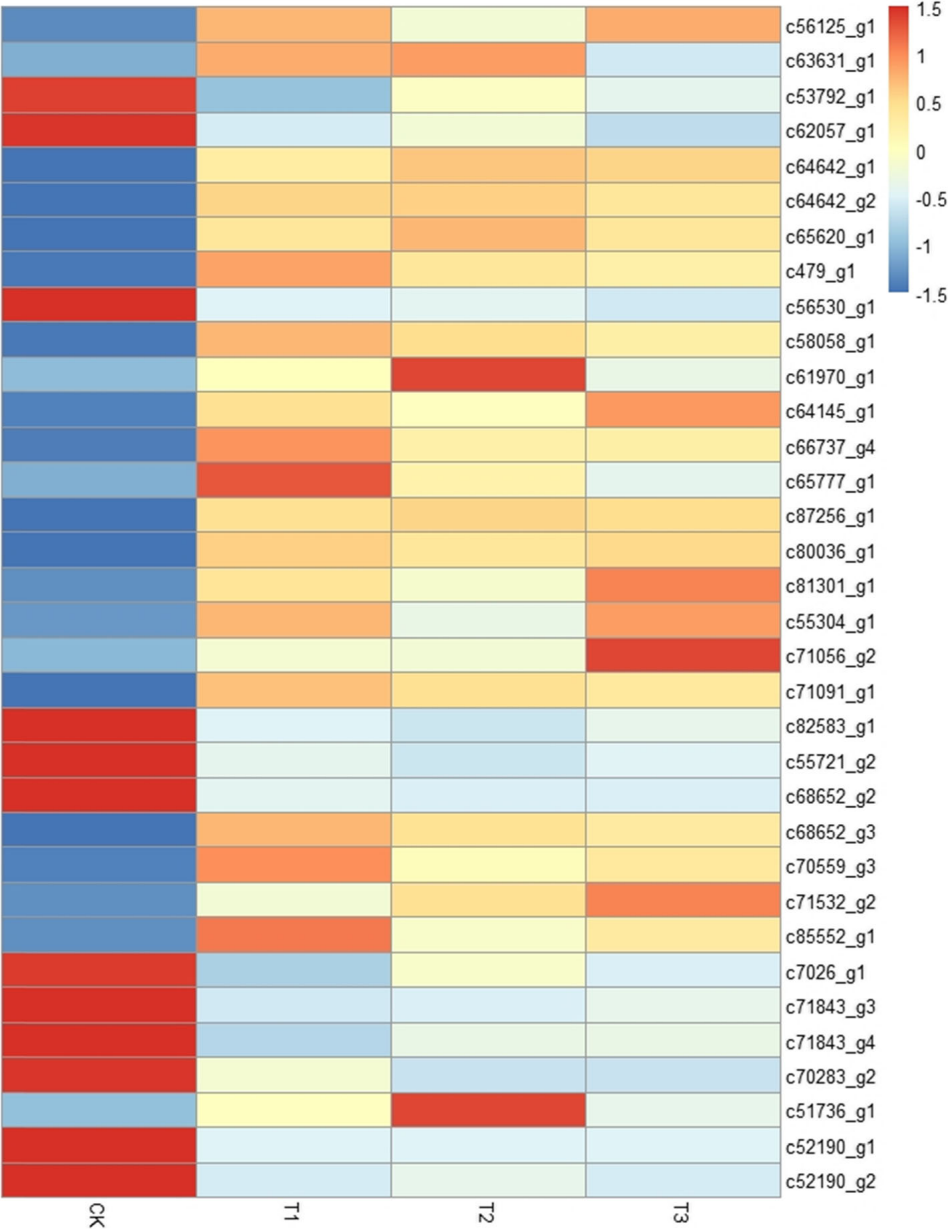
Heat map of DEGs involved in cold signal recognition and transmission in *P. koraiensis* under different cold stress time

The perception and transmission of cold signals is crucial for plants during cold stress. A total of 34 unigenes involved in the perception and transmission of cold signals were significantly differentially expressed (22 up- and 12 downregulated) under cold stress (Fig. 5). Of the 34 cold signal related genes, 31 (21 upregulated and 10 downregulated) acted as calcium ion receptors, 3 (1 upregulated and 2 downregulated) were involved in ABA synthesis and binding. The calcium ion receptor genes mainly encoded annexin, alpha-amylase, calcium-binding protein, calmodulin, calcium-dependent protein kinase, calcineurin B-like protein (CBL), CBL-interacting serine/threonine-protein kinase, CBL-interacting protein kinase and mitogen-activated protein kinase (Additional file 5).

The expression levels of four genes, including annexin D1 (c63631_g1), alpha-amylase isozyme 3C (c65620_g1), calcium-binding protein KIC (c61970_g1) and abscisic acid 8′ -hydroxylase 1 (c51736_g1), first increased significantly and then decreased significantly with the extension of stress time (Fig. 5, Additional file 5).

### Transcription factors (TFs) in response to cold stress in *P. koraiensis*

**Fig. 6:**
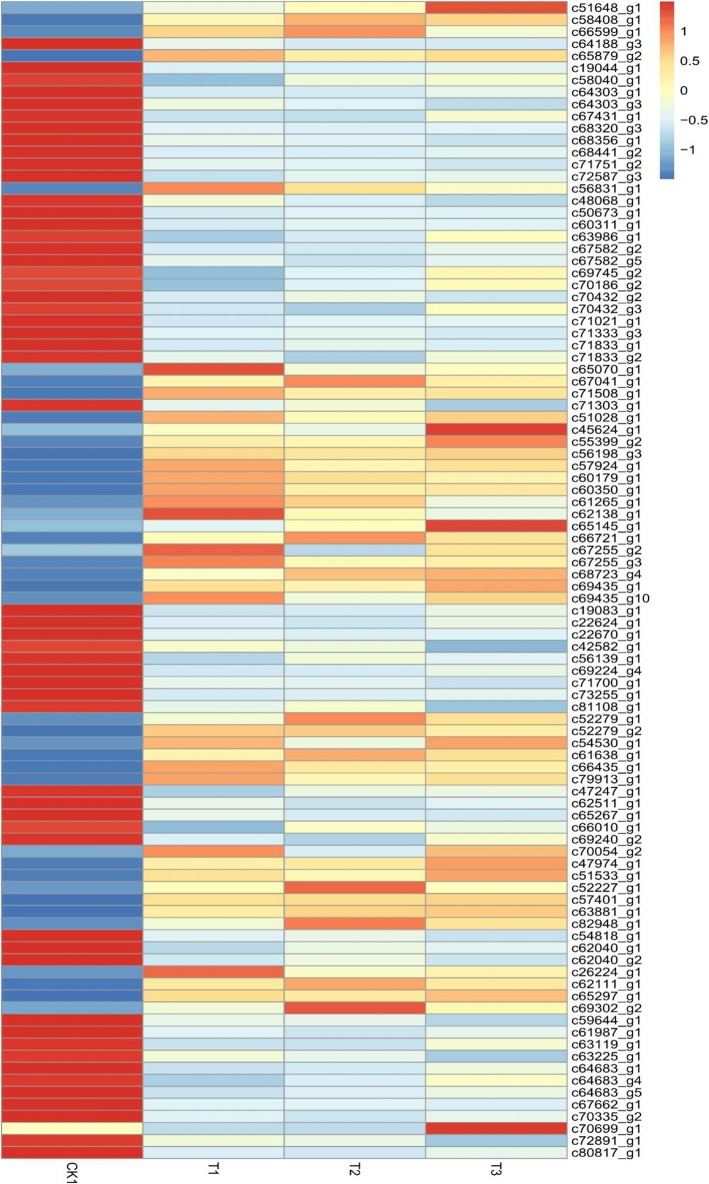
Heat map of DEGs encoding transcriptation factors in *P. koraiensis* under different cold stress time

To explore the TFs in response to cold stress in *P. koraiensis*, 8 TFs were identified, including 96 DEGs (41 upregulated and 55 downregulated) (Fig. 6). Among them, the number of genes for ethylene responsive factor (AP2) was the highest, with 30 genes (5 upregulated and 25 downregulated), followed by MYB with 24 genes (15 upregulated and 9 downregulated), NAM, ATAF1, ATAF2 and CUC2 (NAC) with 16 genes (4 upregulated and 12 downregulated) and zinc finger protein (ZFP) with 11 genes (6 upregulated and 5 downregulated). The number of other transcription factors was small, with 9 basic Helix-loop-helix (bHLH) genes (6 upregulated and 3 downregulated), 4 WRKY genes (3 upregulated and 1 downregulated), 1 upregulated ethylene-insensitive 3 (EIN3) gene and 1 upregulated vascular plant one any zinc-finger protein (VOZ) gene (Additional file 5).

The expression level of the same gene was different for different cold-stress periods. The expression of c58408_g1 and c66599_g1, two *DREB* genes first increased significantly and then decreased significantly with the extension of stress time (Fig. 6, Additional file 5).

### Antioxidant enzymes and antioxidants in response to cold stress in *P. koraiensis*

**Fig. 7:**
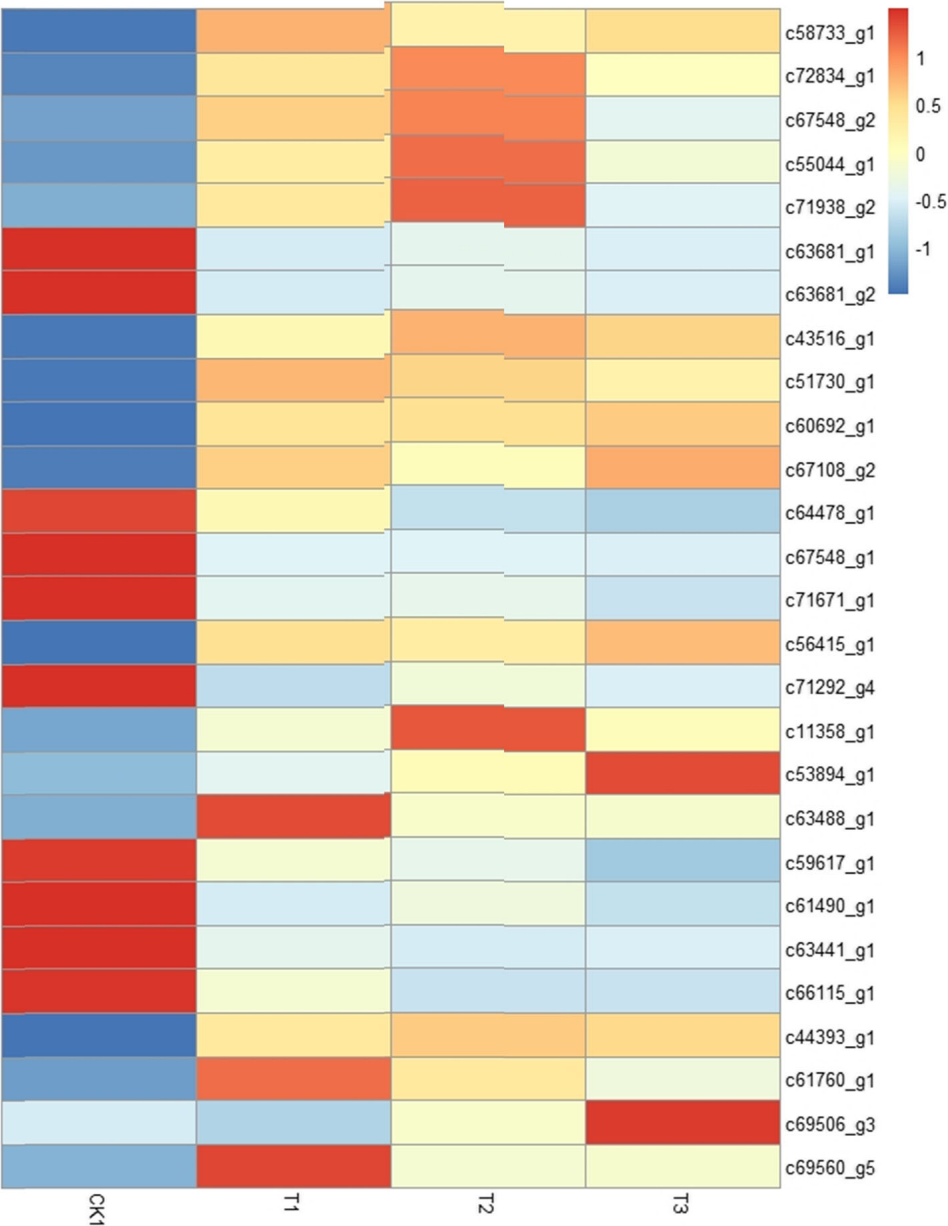
Heat map of DEGs involved in antioxidation mechanisms in *P. koraiensis* under different cold stress time

In this study, 27 DEGs (17 upregulated and 10 downregulated) are shown in Fig.7, which were associated with the reactive oxygen species (ROS) family. These components of the ROS family were antioxidant enzymes and antioxidants including ascorbate peroxidase (APX), superoxide dismutase (SOD), catalase (CAT), glutathione peroxidase (GPX), blue copper protein, glutaredoxin and heat shock protein. The expression level of the same gene was different under different cold stress time. It was noteworthy that the expression level of some genes first increased significantly and then decreased significantly with the extension of stress time. These genes included c67548_g2 (SOD), c55044_g1 (CAT), c71938_g2 (CAT) and c11358_g1 (GPX) (Fig. 7, Additional file 5).

## Discussion

### The general molecular regulation in response to cold stress for plants

**Fig. 8:**
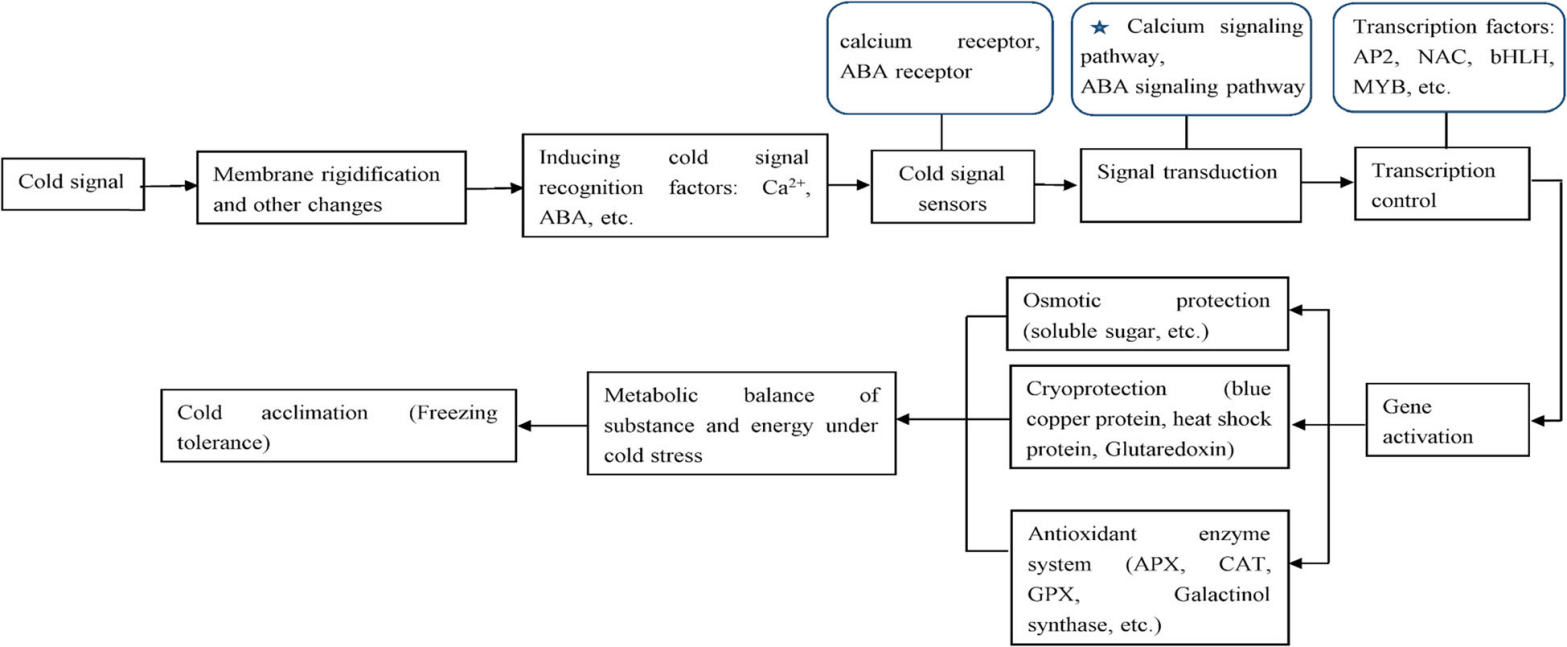
Diagram showing the general molecular regulation mechanism of plants in response to cold stress

The molecular regulation of plants against cold stress was showed in Fig. 8. When plants are subjected to cold stress, the cell membrane first sensed the cold signal with membrane rigidification and other changes, inducing second messengers (Ca^2+^, ABA, etc.) accumulation to transmit cold signals downstream [28]. Calcium receptor is the most important cold signal sensor, and the calcium signaling pathway is the most important signal transduction pathway [28, 29]. Some of the downstream cold signals directly activate the expression of target genes and the others induce the transcriptional regulatory network to activate the expression of a series of cold-related genes, and thus synthesize antioxidant enzymes, condensate protective substances and osmotic regulatory substances, etc. [28]. These substances work together to regulate the metabolic balance of substances and energy, and ultimately resist or adampt cold stress.

### Perception and transmission of cold signals

Woody plants also first show cold signal recognition and transmission in response to cold stress [29]. In addition to the very important calcium ion signal conduction pathway, there are some other conduction pathways, which are mainly related to ABA [30].

In this study, 31 DEGs were found to be related to calcium ion binding and were calcium ion receptors, among which 21 were up-regulated under cryogenic treatment, while only 3 DEGs were related to ABA. It was preliminarily speculated that calcium ion signaling pathway was also the main pathway of intracellular cryogenic signal transduction in *P. koraiensis*. There exist calcium ion receptors in plants, mainly including calmodulins (CaMs), calc-dependent protein kinases (DPKs), calcineurin B-like proteins (CBLs) and CBL-interacting protein kinases (CIPKs) [31, 32], which bind to calcium ions, thus changing the conformation of the proteins and regulating cold-related gene expression or directly transmitting cold signals to the downstream target gene [33, 34]. The expression of the above-mentioned calcium ion receptor genes in *P. koraiensis* was significantly changed under low temperature stress.

Annexin is a calcium-dependent phospholipid binding protein found in plants and animals. It is involved in many life activities regulated by calcium ions, such as signal transduction and calcium ion channel formation, and participates in cold-resistance reactions [35]. Four kinds of annexin were found in cold-resistant wheat, among which, annexin P39 and P225 might be signal transducers in the cold signal transduction or regulators of cytoplasmic calcium ion concentration [36]. 2 upregulated annexin genes (c56125_g1, c63631_g1) were involved in calcium-ion binding to alleviate cold stress in *P. koraiensis*, and similar results were also observed in studies on rice at low temperature [37]. Alpha-amylase can participate in the binding of calcium ions to regulate the recognition and transmission of cold signals to alleviate cold stress. It can also catalyze starch into various soluble sugars, including dextrin, oligosaccharides, a small amount of maltose and glucose, etc. to regulate cell osmotic pressure to alleviate cold stress [38]. Low temperature induced the expression of 3 alpha-amylase genes in *P. koraiensis*, and the expression first increased significantly and then decreased significantly with the extension of low temperature stress time. These results support the previous experiment by the team, in which the content of soluble sugar increased significantly at − 20 °C and a long stress time induced the soluble sugar content to increase first and then decrease [39].

### Transcription factors in response to cold stress

In order to survive, plants form complex and efficient regulatory networks to resist and adapt to cold stress, in which transcriptional regulation plays a key role. Transcription factors regulate the expression of a series of genes and play a key role in plant abiotic adversity response networks by binding to cis-acting elements in the promoter region.

AP2/ERF is a large gene family, which is divided into four subfamilies, namely, AP2, RAV, DREB and ERF [40]. Among them, DREB and ERF are closely related to cold stress, and DREB1/CBF (C-repeat binding factor) plays a role of molecular switch in transcriptional regulation [40, 41]. There were 4 DREB DEGs (c51648_g1 named DREB3A, c58408_g1 named DREB3B, c66599_g1 named DREB3C, c64188_g3 named DREB3D) and 12 ERF DEGS all belong to the AP2 family in this study, which could serve as candidate genes for studying the cold response of conifer species. The expression of DREB3A-C significantly increased with the decrease of the temperature, and their expression were up-regulated by 9.1, 8.1 and 5.6 times at − 20 °C for 6 h, respectively. The expression of DREB3D was down-regulated at − 20 °C. CBF gene family of *Arabidopsis* also included 4 genes, which were CBF1–4, respectively. When CBF overexpression in response to cold, most genes were positively regulated, but a few genes were inhibited by CBF overexpression [42]. Whether DREB3A-D in *P. koraiensis* had similar function need further study.

Studies have shown that MYB15 in *Arabidopsis* was an R2R3 transcription factor and that overexpression of MYB15 resulted in decreased expression of the CBF gene and negative regulation of plant cold resistance [43]. Both c45624_g1 and c65145_g1 in *P. koraiensis* were R2R3 MYB transcription factors, but their expression was up-regulated under cold stress, so further studies were needed. MYB96 has been reported to positively regulate plant cold resistance [44]. Therefore, the regulation of MYB is a complex process under cold stress. NAC and bHLH TFs can be induced by low temperature, and they play important roles in plant cryogenic regulation network; the overexpression of *PbeNAC1* and *PtrbHLH* could improve the cold resistance of *Pyrus betulaefolia* and *Nicotiana tabacum*, respectively [45, 46]. Four NAC and six bHLH DEGs were induced in *P. koraiensis* under cold stress .

In addition to the AP2/ERF, MYB, NAC and bHLH TFs, the DEGs encoding the remaining 4 TFs (WRKY, EIN3, ZFP and VOZ) were also identified in *P. koraiensis* in response to cold stress, which suggested that these TFs also might be important regulators that triggered a cascade of downstream gene expression. However, little research had been conducted on the function of these TFs, and further study of function characterization involved in cold tolerance in plants is still be needed.

### Antioxidation mechanisms in response to cold stress

Hypothermia injury is mainly caused by oxidative stress caused by ROS accumulation. The antioxidant defense mechanism can remove excess ROS to protect plants from the harm for cold-tolerant plants [47]. The antioxidant defense mechanism consists of antioxidant enzymes and antioxidants.

SOD is the first enzyme in antioxidant action. Its main function is to remove O^2−^ and produce H_2_O_2_ at the same time. CAT can degrade excess H_2_O_2_ and other ROS by enzymatic action [48]. APX is one of the key enzymes for H_2_O_2_ clearance, mainly existing in chloroplasts, where CAT enzyme does not exist. Therefore, APX is the key enzyme for H_2_O_2_ clearance in chloroplasts [49]. GPX is a kind of peroxidase containing sulfhydryl, which can remove peroxides such as H_2_O_2_ in plants, so as to avoid ROS damage to plants [50]. Therefore, SOD, CAT, APX and GPX are important antioxidant enzymes in plants. Their high expression can improve the antioxidant capacity of plants and enhance their low temperature tolerance. The lower the temperature, the higher the expression [51, 52]. There were some significantly differentially expressed SOD, CAT, APX and GPX genes in *P. koraiensis* under cold stress, among which the up-regulated genes played an important role. Blue copper protein, glutaredoxin and heat shock protein are all antioxidant substances with antioxidant functions [53, 54]. The expressions of c67548_g2 (SOD), c55044_g1 (CAT), c71938_g2 (CAT) and c11358_g1 (GPX) first increased significantly and then decreased significantly with the extension of cold stress time, which indicated that the longer the low-temperature stress time was, the more seriously the plant was hurt, and the higher the expression of antioxidant enzymes was. However, when the stress time reached a certain level, the plant might adapt to this stress state and the expression level decreased [55]. In the previous report on the physiological indices of *P. koraiensis* seedlings, low temperature (− 20 °C) enhanced the activity of SOD and CAT, and SOD activity increased first and then decreased with the extension of stress time [39], which further demonstrated that low temperature stress could be resisted by increasing the expression of antioxidant enzymes genes, thereby increasing the activity of antioxidant enzymes in *P. koraiensis*.

## Conclusions

In this study, a *Pinus koraiensis* dataset comprising 123,445 unigenes was generated by high-throughput sequencing, and the dynamic changes in gene expression were observed under cold stress for different stress times. A large number of DEGs were identified, especially the DEGs involved in the perception and transmission of cold signals, DEGs encoding TFs related to cold regulation and the DEGs removing ROS in antioxidation mechanisms. The transcriptome and digital expression profiling of *P. koraiensis* could facilitate the understanding of the molecular control mechanism related to cold responses and provide the basis for the molecular breeding of conifers. The DEGs of *P. koraiensis* without annotation in the Nr database were what we would study in the future, which might be specific to *P. koraiensis*.

## Methods

### Plant materials and cold treatments

Seeds of *P. koraiensis* were obtained from Kaishan village seeds orchard (129°45′ E, 42°40′ N) located on the east side of Changbai Mountain in Longjing city, northeast China. The average annual temperature, temperature in January and temperature in July are 5.3 °C, − 13.4 °C and 21.2 °C, respectively [56]. The lowest temperature in Longjing was − 34.8 °C [2]. The seeds were obtained in 2010 and germinated in 2011 in a greenhouse. After 4 years, 300 healthy seedlings were taken back to the laboratory growing in the greenhouse (20 °C, air humidity from 50% to 65%, 16 h/d light cycle, light intensity 150 μmol·m^− 2^·s^− 1^) for 1 month until they fully adapted to the environment. For cold treatment, 120 well-performed and similar seedlings were immediately placed at − 20 °C extreme low temperature from the ambient temperature (20 °C, control (CK)), and the needles were harvested after 0 (CK), 6, 24 or 48 h, which was achieved by the freezer Haier BC/BD-318HD (10 °C ~ − 26 °C). For each time point, the obtained needles included three biological replicates, and each replicate was a mixture from 10 independent seedlings.

### RNA extraction

Total RNA was extracted using the plant total RNA extraction kit (TaKaRa, Beijing, China) by following the detailed instructions from the manual. The total RNA extracted was detected by a biological analyzer (2100, Agilent, USA). Next, 20 μg of high-quality RNA was selected from each of the twelve RNA samples (each treatment including three biological replicates) for the construction of cDNA libraries, and the remaining high-quality RNA was used for qRT-PCR analysis.

### cDNA library construction and RNA-sequencing

mRNA was enriched using magnetic beads with Oligo (dT), and fragmentation buffer was added to break the mRNA into short fragments (200–700 bp). The short mRNA fragments were used to synthesize double-chain cDNAs using random hexamer primers, buffer, dNTPs, RNase H and polymerase I. The cDNAs were purified using a PCR purification kit (QiaQuick, USA) and were washed with EB buffer for terminal repairing and poly(A) addition. Different cDNA fragments with various sizes were separated via agarose gel electrophoresis and were enriched via PCR amplification to construct cDNA libraries. The obtained 12 cDNA libraries were sequenced by DNA sequencer (HiSeq™ 2000, Illumina, USA), and the sequencing strategy was PE150.

### Data filtering and de novo assembly

Data filtering was performed through the following steps for the obtained reads by sequencing: first, adaptor-containing reads were removed; second, the reads containing more than 5% ambiguous nucleotides were removed; and finally, the low-quality reads that contained more than 15% bases with Q-value ≤19 were removed, and the clean reads were obtained for de novo assembly. Trinity was the assembly software used, which linked the reads with overlapping sequences into much longer contiguous sequences; these longer sequences are termed as ‘contigs (contiguous sequences)’. Next, the reads were compared back to the contigs. The distance between different contigs from the same transcript was determined according to paired-end reads. The contigs were assembled by Trinity to obtain the unigene sequences that could not be extended at both ends.

All the nonredundant unigenes were subjected to BLASTn or BLASTx alignment (*E-*value < 10^− 5^) against nucleic acid database Nt or various public protein databases that included Nr, UniProt, KEGG and COG. The proteins with the best comparison result were selected to determine the sequence direction of the unigenes. If the comparison results were inconsistent, the sequence direction of the unigenes was determined based on the priority of Nr, UniProt, KEGG and COG. The ESTScan software was used to determine the sequence direction of the unigenes when the above databases were not suitable for alignment [57].

### Sequence annotation and analysis of DEGs

The Blast2GO software was used to generate the GO terms based on the Nr annotation for the nonredundant unigenes [26], and the KEGG database was used to determine metabolic pathways of the unigenes [58]. Twelve cDNA libraries of *P. koraiensis* that was treated for 0, 6, 24, and 48 h at − 20 °C were sequenced, and the raw data were analyzed via the above methods. The expression of unigenes was calculated by RPKM, which can eliminate the influence on the expression of calculated genes [25]. The calculated gene expression level was directly used to compare the differences in gene expression between different cDNA libraries. To determine significant differences, a false discovery rate < 0.001 and an absolute value of log_2_FoldChange > 1 were set as thresholds. Unigenes with different RPKM values and conforming to thresholds were identified as DEGs [27] . The DEGs mentioned in this paper are all significant. Next, GO function analysis and KEGG Pathway analysis were performed on DEGs [58, 59].

### qRT-PCR test

The twelve remaining total RNA samples were reverse-transcribed with the ReverTre Ace®qPCR RT Kit (Toyobo, Osaka, Japan). The reverse transcription reaction contained 1 μg of RNA, 2 μl of 5 × RT Buffer, 0.5 μl of Primer Mix, 0.5 μl of Enzyme Mix and deionized water in a final volume of 10 μl; the reaction was conducted at 37 °C for 15 min and 98 °C for 5 min. Each of the generated cDNAs was diluted 10 times as the qRT-PCR template. qRT-PCR was performed with a DNA Engine Opticon™ 2 Real-Time System (Bio-Rad, USA), and the reaction was composed of 10 μl of 2 × SYBR Green Realtime PCR Master mix (Toyobo, Osaka, Japan), 2.5 μl of cDNA, 0.5 μl of upstream primer, 0.5 μl of downstream primer and deionized water in a final volume of 20 μl. Meanwhile, the PCR was conducted at 94 °C for 30 s, followed by 45 cycles of 94 °C for 12 s, 54 °C for 30 s and 72 °C for 30 s. The expression level of the selected genes was determined by the 2^-ΔΔCt^ algorithm, and the *P. koraiensis* Tubulin alpha (TUBA) gene was used as an internal control [60]. Each sample had three biological replicates, and the data were presented as the means ± standard errors (SE) (*n* = 3). The primer sequences of the selected genes are listed in Table 3.

## Supplementary information

**Additional file 1. **Go analysis of the unigenes for *P. koraiensis* in response to cold stress.

**Additional file 2. **COG annotation of the unigenes for *P. koraiensis* in response to cold stress.

**Additional file 3.** Gene Ontology clustering of DEGs.

**Additional file 4.** KEGG analysis of the DEGs.

**Additional file 5.** All the DEGs in each comparison.

## Data Availability

The datasets used and/or analysed during the current study are available from the in the US National Library of Medicine, https://www.ncbi.nlm.nih.gov/bioproject/PRJNA510863.

## Abbreviations

ABA: Abscisic acid
AP2: Ethylene responsive factor
APX: Ascorbate peroxidase
bHLH: Basic Helix-loop-helix
CaMs: Calmodulins
CAT: Catalase
CBF: C-repeat (CRT)-binding factors
CBLs: Calcineurin B-like proteins
CIPKs: CBL-interacting protein kinases
COG: The Clusters of Orthologous Groups of proteins database
COR: Cold-regulated
DEGs: Differential expressed genes
DPKs: Calc-dependent protein kinases
DREB: Dehydration-responsive element-binding factors
eggNOG: The evolutionary genealogy of genes: Nonsupervised Orthologous Groups database
EIN3: Ethylene-insensitive 3
GLR: Glutaredoxin
GO: The Gene Ontology database
GPX: Glutathione peroxidase
HOS: High Expression of Osmotically Responsive Gene
ICE: Inducer of CBF Expression
KEGG: Kyoto Encyclopedia of Genes and Genomes database
MYB: Myeloblastosis
NAC: NAM, ATAF1, ATAF2 and CUC2
Nr: NCBI nonredundant database
Nt: NCBI Nucleotide sequence database
PE: Paired-end
Pfam: The Protein families database
qRT-PCR: Quantitative real-time PCR
ROS: Reactive oxygen species
RPKM: Reads per kilobase per million mapped reads
SOD: Superoxide dismutase
TFs: Transcription factors
UniProt: The Universal protein database
VOZ: Vascular plant one any zinc-finger protein
ZFP: Zinc finger protein
